# Medication prescribing for asthma and COPD: a register-based cross-sectional study in Swedish primary care

**DOI:** 10.1186/1471-2296-15-54

**Published:** 2014-03-25

**Authors:** Paolina Weidinger, J Lars G Nilsson, Ulf Lindblad

**Affiliations:** 1 Department of Public Health and Community Medicine/Primary Health Care, The Sahlgrenska Academy at Gothenburg University, Gothenburg, Sweden; 2 Skaraborg Primary Care Research and Development Unit, Skövde, Sweden; 3 Skaraborg Institute, Skövde, Sweden

**Keywords:** Asthma, COPD, Prescribing, Off-label, Guidelines, Cross-sectional study

## Abstract

**Background:**

There is a gap between prescribed asthma medication and diagnosed asthma in children and adolescents. However, few studies have explored this issue among adults, where asthma medication is also used for the treatment of chronic obstructive pulmonary disease (COPD). The aim of this study was to examine the relationship between prescribing of medications indicated for asthma and COPD and the recorded diagnosis for these conditions.

**Method:**

In a register-based study, individuals prescribed a medication indicated for asthma and COPD during 2004-2005 (Group A; n = 14 101) and patients with diagnoses of asthma or COPD recorded during 2000-2005 (Group B; n = 12 328) were identified from primary health care centers in Skaraborg, Sweden. From a 5% random sample of the medication users (n = 670), the written medical records were accessed. Primary outcomes: prevalence of medication and diagnoses, reasons for prescription. Secondary outcomes: type and number of prescribed drugs and performance of peak expiratory flow or spirometry.

**Results:**

Medications indicated for asthma and COPD was prescribed to 5.6% of the population in primary care (n = 14 101). Among them, an asthma diagnosis was recorded for 5876 individuals (42%), 1116 (8%) were diagnosed with COPD and 545 (4%) had both diagnoses. The remaining 6564 individuals (46%) were lacking a recorded diagnosis. The gap between diagnosis and medication was present in all age-groups. Medication was used as a diagnostic tool among 30% of the undiagnosed patients and prescribed off-label for 54%. Missed recording of ICD-codes for existing asthma or COPD accounted for 16%.

**Conclusion:**

There was a large discrepancy between prescribing of medication and the prevalence of diagnosed asthma and COPD. Consequently, the prevalence of prescriptions of medications indicated for asthma and COPD should not be used to estimate the prevalence of these conditions. Medication was used both as a diagnostic tool and in an off-label manner. Therefore, the prescribing of medications for asthma and COPD does not adhere to national clinical guidelines. More efforts should be made to improve the prescribing of medication indicated for asthma and COPD so that they align with current guidelines.

## Background

Asthma and chronic obstructive pulmonary disease (COPD) are respiratory diseases representing a major burden in health care [1,2]. The prevalence varies between countries, with an estimated prevalence of 8% for asthma, and approximately 4-6% of the adult population having COPD in Sweden [3,4]. There are a number of national and international guidelines for the diagnosis and management of asthma and COPD [2,5,6]. Asthma medication is approved for use in both asthma and in COPD and for COPD there is also specific treatment, such as anticholinergics for inhalation, not approved for use in asthma [6].

The diagnosis and management of asthma and COPD often stray from guidelines [7,8] leading to both under-diagnosis, misdiagnosis and inappropriate prescribing practices [9-11]. Particularly for children, asthma medication is often prescribed without diagnosed asthma [12-15]. One third of all preschool children experience periods of wheezing [14] and it may not be easy to differentiate asthma from other wheezing disorders. In the absence of objective measurement, asthma medication is often used as diagnostic tool [15]. However, for patients from the age of six years; the diagnosis can be made with reasonable certainty based on objective measurements [3]. Thus the discrepancy between prescribed asthma medication and diagnosed asthma can potentially be reduced from the age of six. However, in the Netherlands, Zuidgeest et al. found that the gap also continues into adolescent age and they conclude that this could be due to factors such as under-diagnosis, off-label prescribing and the use of asthma medications as a diagnostic tool [12]. Few studies have explored the discrepancy between prescribed medication and diagnosed asthma or COPD in adults. In the Netherlands, Lucas et al. [16] found that only 74% of the prescriptions of inhaled corticosteroids were prescribed to patients actually having asthma or COPD, indicating a discrepancy also between prescribed medication and COPD. The use of prescribed medication as a tool in the diagnostic assessment was shown in a study from Germany [8]. In this study, trial of medication was used in 22% of the patients with asthma, but among the patients with COPD, the number was only 5%. Trial of medication thus appears to exist in both asthma and COPD, but to a different extent.

Off-label prescribing is common in many diseases [13,17]. In such prescribing, a registered medicine is used in a manner not comprised in the license, including the use of drugs in non-approved age-groups (i.e. children), unrecommended doses, formulations or indications [18]. In the US, 40% of the medications for asthma are prescribed off-label [17]. Even though not recommended, for some diagnoses it may sometimes be clinically appropriate and might reflect innovative clinical practice. However, in a US study, 73% of the off-label prescriptions had no or little scientific support [18], and were associated with a number of safety, clinical and ethical issues. Published reports thus indicate that there is a gap between prescribed asthma medication and physician-diagnosed asthma among children and adolescents. However, only few studies have explored this discrepancy among adults, where asthma medication is also indicated for the treatment of COPD.

### Aim

The aim of this study was to examine the relationship between prescribing of medications indicated for asthma and COPD and the recorded diagnosis for these conditions.

## Methods

### Setting

The county of Skaraborg, Region Västra Götaland, in southwest Sweden (approximately 260 000 inhabitants) has a well established primary health care with 24 primary health care centres (PHC) covering 97% (n = 251 718) of the population during the time of the study. Since 1998, it is possible to extract information from the computerized medical records collected during patient visits, including diagnostic measurements, prescribed medications and the physicians’ written notes. This information has previously been used in register-based studies [7,19].

### Study population

To be able to determine to what extent prescribing was based on diagnoses and if diagnoses correlated to prescribing, two different patient groups, A and B, were selected. To be able to access information only available in the medical records, a random sample was extracted and the medical records were manually examined for these patients.

#### Group A (selected based on prescribing)

Data on all individuals with at least one prescription of medication indicated for asthma or COPD issued during 2004-2005. Medications were identified by their respective ATC-codes (Anathomical Therapeutic Chemical classification) [20]. Included drugs were limited to those used in both asthma and COPD; short-acting ß_2_-adrenergics (SABA; R03AC02 and R03AC03), long-acting ß_2_-adrenergics (LABA; R03AC12 and R03AC13), inhaled corticosteroids (ICS; R03BA01, R03BA02, R03BA05 and R03BA07), and fixed combinations of long-acting ß_2_-adrenergics and inhaled corticosteroids (R03AK06 and R03AK07). Drugs exclusively used in COPD belonging to the R03BB group (anticholinergics) were not included.

#### Group B (selected based on diagnoses)

Data on patients with recorded diagnoses of asthma or COPD, during the period of 2000-2005, with at least one subsequent contact during 2004-2005. The ICD-codes are used in Swedish primary care and ICD codes J45 and J44 were used to identify patients with asthma and COPD respectively. The total target population from group A and B was 18 892 individuals.

#### Random sample

**Figure 1:**
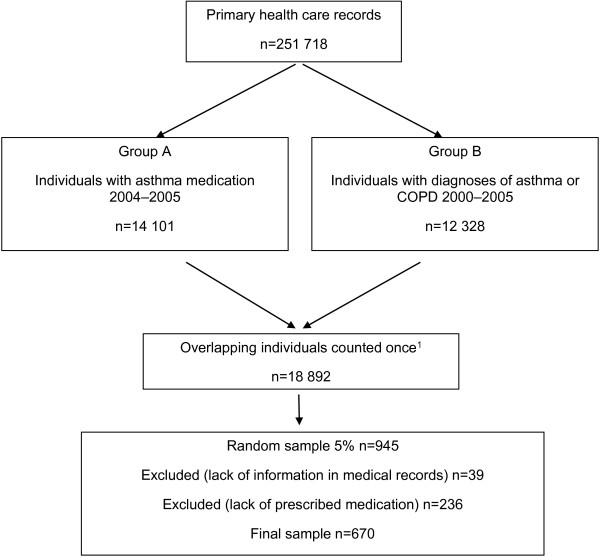
Schematic outline of the patient selection in the study.

The random sample of 5% (n = 945) was randomly extracted from Group A and B using a computer-based tool; Statistics Package for Social Sciences (SPSS version 19.0 for PC), and then categorized into age-groups. A number of 39 individuals were excluded due to insufficient information in their medical records and 236 individuals due to lack of prescribed medication (originated only from selection-Group B). The final sample for medical record review thus consisted of 670 individuals. A schematic outline of the patient selection is presented in Figure 1.

Regional Ethics Review Board at Gothenburg University (Dnr. 191-07) approved the study protocol.

### Analysis

The primary outcomes were the difference between prevalence of prescribed medication and prevalence of recorded diagnoses, recorded ICD-code or indication based on written note when no ICD-code was given, including symptom-based indications. Secondary outcomes were type of prescribed drug, number of prescriptions and performed peak expiratory flow or spirometry. The random sample was checked for representativeness against the data set of the total selected population concerning variables available in both data sets, such as key demographics and clinical factors, and a close resemblance was found. In the random sample, the patient records were reviewed in detail and reasons for prescription were categorized into:

•*Asthma recorded diagnosis*: ICD-code of asthma recorded.

•*COPD recorded diagnoses:* ICD-code of COPD. Patients with both COPD and asthma were included in this group.

•*Provisional diagnoses:* Diagnosis of asthma or COPD based on written notes in the text part of the medical records.

•*Trial of medication*: The physician was uncertain about the diagnosis, to be confirmed by response to medication as a part of the diagnostic assessment.

•*Off-label*: Off-label by indication; the medication was prescribed for other non-recommended indications or there was no information in the medical record of the patient having neither asthma nor COPD. Off-label by age, dosage, duration of time or route of administration were not considered.

A prescription issued in Sweden is valid for one year, and for long-term medication, prescriptions are commonly issued for the entire year of treatment. SABA used during post-bronchodilator spirometry is provided by the care-giver and are not prescribed to the patient for this occasion.

### Statistical methods

Descriptive statistics were performed using Statistics Package for Social Sciences (SPSS version 19.0 for PC). The data was presented as absolute numbers and percentages and the statistical significance of differences between groups were assessed using chi-squared tests with a significance threshold of p < 0.05. All tests were two-sided.

## Results

Characteristics of the population in Skaraborg, the selected population from the PHCs and the random sample is presented in Table 1.

**Table 1:** Characteristics of the population in Skaraborg, the selected population with medication or diagnoses in primary care, and the random sample

	**Skaraborg population**	**Group A**	**Group B**	**Random sample**
		**Medication users**	**Asthma or COPD diagnoses**	
	**n = 255 382**	**n = 14 101**	**n = 12 328**	**n = 670**
	**n (%)**	**n (%)**	**n (%)**	**n (%)**
*Gender*				
Male	127 470 (50)	5 970 (42)	5 527 (45)	295 (44)
Female	127 912 (50)	8 131 (58)	6 801 (55)	375 (56)
*Age (y)*				
0–17	54 536 (21)	2 118 (15)	2 103 (17)	100 (15)
18–70	166 315 (65)	9 488 (67)	7 420 (60)	452 (68)
≥71	34 531 (14)	2 495 (18)	2 805 (23)	118 (18)

Note; Groups A and B are not mutually exclusive.

During 2004-2005 medication indicated for asthma and COPD were prescribed to 5.6% (Group A; n = 14 101) of the individuals in Skaraborg primary care. Within this population of medication users, an asthma diagnosis was recorded for 5876 individuals (42%), 1116 (8%) were diagnosed with COPD and 545 (4%) with both COPD and asthma. The remaining 6564 individuals (46%) were lacking a recorded ICD-code for any of the diagnoses.

**Figure 2:**
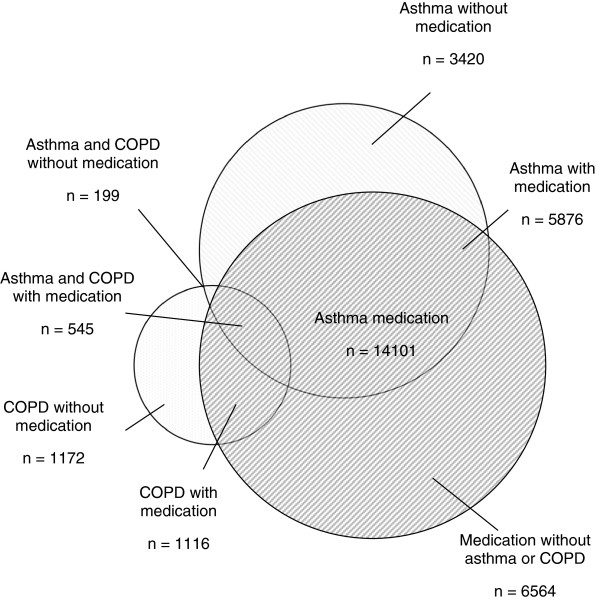
Proportional Venn-diagram of the medication users and their recorded diagnoses.

The results from Group B demonstrated that an asthma diagnosis was recorded for totally 9296 individuals, 2288 had COPD and 744 had both asthma and COPD. Prescription of medication were lacking among 3420 of the individuals with asthma (37%), 1172 with COPD (51%) and among 199 with both asthma and COPD (27%). The prevalence of asthma and COPD based solely on recorded diagnosis was for the Skaraborg population 3.6% and 1.2% respectively. The lack of congruence between medication users and individuals with a recorded diagnosis of asthma or COPD is presented graphically in a Venn diagram in Figure 2.

**Figure 3:**
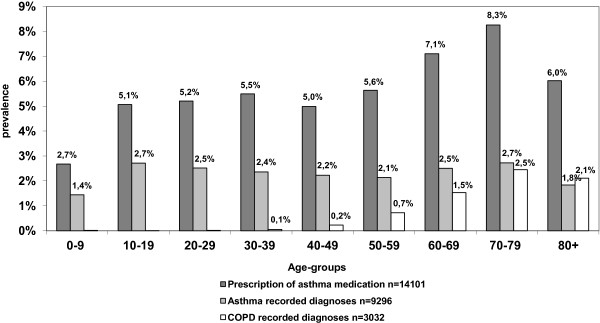
**Prevalence of medication in relation to prevalence of recorded diagnosis in different age-groups.** Lower rate of diagnoses than prescriptions was apparent in all age-groups. Patients with both asthma and COPD (n = 744) are included in the COPD-group.

The prevalence of medication in relation to the prevalence of recorded diagnoses, in different age-groups is presented in Figure 3. Lower rate of diagnoses than prescriptions was apparent in all age-groups. Prevalence of medication use was lowest among the youngest patients and increased with age. The average discrepancy (calculated as a weighted mean) between medication prescribing and diagnosis was 2.5 percentage points.

### Random sample

The random sample comprised of 670 individuals. The detailed analysis is presented in Tables 2 and 3. Records of diagnoses were found for 391 of 670 patients (58%), 320 with asthma and 71 with COPD. In total, 279 patients (42%) therefore lacked a recommended diagnosis for prescribing of medication. However, among these individuals 44 (16%) had a provisional diagnosis (asthma or COPD based on doctor’s written notes). Among the remaining individuals, 85 (30%) were prescribed medication on trial as a part of the diagnostic assessment and 150 (54%) individuals were prescribed off-label. Although there appeared to be differences in prescribing by sex, a chi-squared test to assess differences in prescribing between men and women was not statistically significant (p = 0.061). The most common indications for off-label prescribing were diagnoses of cough (n = 18; 12%) and acute bronchitis (n = 16; 11%) or symptoms of cough, airway obstruction and dyspnoea (n = 21; 14%).

**Table 2:** Demographic characteristics of subjects in the random sample stratified by recorded asthma or COPD diagnosis, provisional diagnoses, trial by medication and off-label prescribing

**Treatment**	**Total**	**Recorded asthma diagnosis**	**Recorded COPD diagnosis**	**Provisional diagnoses**	**Trial by medication**	**Off-label prescribing**
	**n**	**n (%)**	**n (%)**	**n (%)**	**n (%)**	**n (%)**
*All patients*	670	320 (48)	71 (11)	44 (7)	85 (13)	150 (22)
*Gender*						
Male	295	140 (44)	36 (51)	24 (55)	39 (46)	56 (37)
Female	375	180 (56)	35 (49)	20 (45)	46 (54)	94 (63)
*Age groups*						
0–17	100	59 (18)		8 (18)	11 (13)	22 (15)
18–70	452	223 (70)	41 (58)	27 (61)	58 (68)	103 (69)
≥71	118	38 (12)	30 (42)	9 (20)	16 (19)	25 (17)

**Table 3:** Random sample; number of prescriptions, therapy groups and clinical evaluation

**Treatment**	**Total**	**Recorded asthma diagnosis**	**Recorded COPD diagnosis**	**Provisional diagnoses**	**Trial by medication**	**Off-label prescribing**
	**n**	**n (%)**	**n (%)**	**n (%)**	**n (%)**	**n (%)**
*All patients*	670	320 (48)	71 (11)	44 (7)	85 (13)	150 (22)
*Number of prescriptions*^1^						
1 prescription	332	135 (42)	22 (31)	25 (57)	34 (40)	116 (77)
2 prescriptions	196	117 (37)	16 (23)	15 (34)	26 (31)	22 (15)
≥ 3 prescriptions	142	68 (21)	33 (46)	4 (9)	25 (29)	12 (8)
*Therapy groups*^2^						
SABA only	207	54 (17)	15 (21)	14 (32)	29 (34)	95 (63)
ICS and SABA	141	83 (26)	7 (10)	13 (30)	24 (28)	14 (9)
ICS and LABA	91	55 (17)	12 (17)	7 (16)	6 (7)	11 (7)
Fixed combination of ICS/LABA	175	102 (32)	34 (48)	8 (18)	17 (20)	14 (9)
ICS only^3^	56	26 (8)	3 (4)	2 (5)	9 (11)	16 (11)
*Clinical evaluation*						
PEF^4^ only	36	25 (8)	1 (1)	1 (2)	2 (2)	7 (5)
Spirometry	207	110 (34)	37 (52)	16 (36)	24 (28)	20 (13)

^1^Number of prescriptions = number of occasions where the patient had one or more medications prescribed.

^2^Therapy groups according to the treatment steps in the therapy recommendations, SABA = Short acting β_2_-agonists; LABA = Long-acting β_2_- agonists; ICS = Inhaled Corticosteroids.

^3^ICS is not recommended as monotherapy.

^4^PEF = Peak Expiratory Flow.

Among patients with asthma, 135 (42%) were issued a medication once during 2004-2005 and 117 (37%) patients were issued medication twice (Table 3). Three or more medications were prescribed amongst 33 out of 71 (46%) patients with COPD. Among individuals prescribed medication off-label, SABA as the only medication was most common as compared to the rest of the medication users (95; 63%, p < 0.001). Table 3 also shows that patients with asthma or COPD diagnoses had a higher rate of prescribing than those without recorded diagnoses. Low levels of PEF and spirometry are shown in Table 3.

## Discussion

This is an extensive survey of drug prescribing among a large broadly representative group of patients and based on individual patient records. Our study indicate that medication indicated for asthma and COPD seems to be both over- and under-used; over-used when only half of the medication users actually had one of the recommended diagnoses of asthma or COPD recorded. Under-used when individuals having asthma or COPD were lacking prescriptions of medication. We also show that the use of asthma medication as a diagnostic tool and off-label prescribing were common. Our results are of interest when auditing prescribing in general practice. It should also be observed by researchers in the field studying the epidemiology of asthma and COPD medication use, as discussed below.

The Venn diagram in Figure 2, shows prescribing of medication in relation to diagnoses of asthma or COPD. The figure illustrates the discrepancy between the prevalence of asthma and COPD and the prescribing of medication indicated for these conditions. A considerable proportion of the individuals with asthma or COPD did not have any medication prescribed during the study period, which likely indicates under-medication [9,21]. It could also be due to previous incorrect diagnoses of asthma or COPD of individuals not having the recorded disease [22,23]. For COPD it could also be due to exclusive prescribing of anticholinergics (specific only for COPD), without simultaneous prescribing of SABA, LABA or ICS. Approximately half of the individuals prescribed a medication had neither a diagnosis of asthma nor of COPD recorded, which probably indicate over-medication (i.e. off-label prescribing) [13,17,24]. It could also be due to under-diagnosis of asthma or COPD [10,11], which is supported by the low prevalence figures of 3.6% and 1.2%, as compared to the national prevalence of 8% and 4%, respectively [25]. Similar figures have previously been reported from other regions of the Swedish primary care [26].

We have shown that the gap between asthma medication and recorded diagnoses, previously shown mostly in children and adolescents [12,13], exists in all age-groups (Figure 3). The average prevalence of medication use was 5.6%, in line with previous findings [12,21]. However, we found that there are fewer children using asthma medication, as compared to the results by Zuidgeest et al. [12] and a decrease in volume of prescribing in the elderly population, which runs counter to the findings of another Swedish study [21]. These differences could be due to inclusion of anticholinergics included in the total volume of drugs in this age-group, in the study by Haupt et al. [21]. It could also be due to hospital doctors’ prescribing medication for both the oldest and the youngest, which are not shown in data from primary care [27], and therefore could be interpreted as an absence of medication.

The detailed examination of patient records in the random sample showed that more than half of the undiagnosed medication users (54%) had the medication prescribed off-label. Many previous studies have been performed concerning off-label prescribing among children [24]. Our study shows that off-label prescribing of asthma medication also is common among adults, and used to treat patients with diagnoses of acute bronchitis and cough. Similar findings were reported for ß_2_-adrenergics for acute bronchitis [28]. Even though the Cochrane group found no evidence for such prescribing [29]; the practice exists, suggesting lack of adherence to evidence based prescribing. The use of medication off-label may be a waste of resources in health care. Moreover, asthma medication represents the most frequently reported cause of adverse reactions among children and adolescents, since half of them were prescribed off-label [30]. Therefore the practice also could constitute a risk for these patients.

We found that asthma medication was often used as a part of the diagnostic assessment (30%) and 16% of the individuals, had provisional diagnoses of asthma or COPD, i.e. asthma or COPD based on doctor’s written notes and no ICD-code recorded (Table 3). Low levels of PEF and spirometry are shown in Table 3, similar results are reported in our previous study [7], indicating low adherence to guidelines. Among the individuals where the medication was used as a part of the diagnostic assessment, the trial of medication may have been used for diagnosis rather than peak expiratory flow measurement or spirometry, as also reported elsewhere [8].

Among patients with asthma, 79% were issued a medication once or twice during the two-year period (Table 3). When also considering those with no prescriptions, we conclude that many patients may be inadequately treated, which has also been shown in other studies [9,10]. For patients with COPD, the situation was different. They had more prescriptions issued (Table 3) and they contributed extensively to the total population of asthma medication users in the older age-groups (Figure 3). Assuming one year’s supply for each prescription (which is common in Sweden) our findings suggest over-medication among patients with COPD, since approximately half of the patients were prescribed three or more medications during the two-year period (Table 3). However, since under-detection of COPD is common [11], the patients detected and diagnosed may be those with more severe COPD and therefore in need of more medication. This interpretation is further supported by the more common use of LABA and fixed combination of LABA/ICS among these patients, and the fact that these medications are to be used in the moderate to severe stages of COPD [6].

Among the 71 patients with COPD, only 37 (52%) had spirometry performed. Related data from USA and Denmark show similar levels [31,32], which has also been reported in our previous study [7]. This is a clear shortfall in the diagnosis of COPD and a lack of clinical resource and training may be a liable factor in this case. However, access to spirometry equipment was reported by 95% of the primary health care centers in the same region during the time of the study [33] and the low levels of spirometry found in the present study are to be explained by other factors than availability of spirometry. Against this background of deficiencies in objective diagnostic measures, a trial of medication to help diagnose asthma and COPD appears to be widespread practice.

The use of asthma medication as a proxy for disease prevalence is an on-going debate. Our study confirms previous results of incongruenty between prescription and diagnosis. In the Netherlands, using data from primary care, a single prescription of asthma medication could identify 95% of adult asthma patients but at the same time 30% of all asthma medication users with a single prescription were non-asthma patients [34]. On the other hand, several studies have shown a close resemblance between prevalence of medication and prevalence of disease [10,21]. However, in these studies the use of non-linked data sets and the lack of information about diagnoses in databases with dispensed medication are limitations. Our study indicates that the prevalence of asthma and COPD cannot be reliably estimated from the prevalence of prescribing of medication indicated for either of the conditions.

### Strengths and limitations of the study

The data we used had good coverage (97%) of the population of Skaraborg. This along with the large sample size, are some of the strengths of our study. The sample size for detailed examination of patient records, was restricted for practical reasons (n = 670). However, the characteristics of our random sample were similar to the whole population of Skaraborg (Table 1) and therefore likely to be representative for the region. Moreover, the manual examination of the information in the free text field of patient records is an important strength in our study. The sample size was not based on a formal power calculation, but rather chosen considering conclusive findings in previous studies of comparable size [8,10]. When using data from routine care, there is a risk that the information about diagnoses and medication has not been documented or appropriately coded in the patients’ records. However, due to the detailed examination of the free text field, this risk should be reduced. In this study we were limited to data from primary care. Data from additional caregivers (such as hospital doctors) managing the patient and prescribing medicines are not included, which could be interpreted as an absence of medication. Also we did not have data on pharmacy dispensing or medication compliance. In both cases there is a risk of overestimation of drug use. Treatment by anticholinergics exclusively used in COPD was not included, which could underestimate the total medication use among the patients with COPD. Our evaluation of off-label medication use is limited by indication. We did not examine off-label prescribing by age, dosage, duration, or route of administration.

## Conclusion

There was a large discrepancy between prescribing of medication and the prevalence of diagnosed asthma and COPD. Consequently, the prevalence of prescriptions of medications indicated for asthma and COPD should not be used to estimate the prevalence of these conditions. Asthma medication was often prescribed outside recommendations and used both as a diagnostic tool and in an off-label manner. Therefore, the prescribing of medications for asthma and COPD currently does not adhere to national clinical guidelines. More efforts should be made to improve the prescribing of medication indicated for asthma and COPD so that they align with current guidelines. We recommend that the prescribing of these medications and the medical management of both asthma and COPD are regularly audited to improve the prescribing practices in Skaraborg.

## Competing interests

We have no conflicts of interest to declare.

## Authors’ contributions

PW coordinated data collection and analysis, wrote the first draft of the paper and prepared the manuscript for publication. JLGN contributed to revisions of the paper and writing the discussion. UL supervised data collection and contributed to revisions of the paper. All authors read and approved the final manuscript.

## Pre-publication history

The pre-publication history for this paper can be accessed here:

http://www.biomedcentral.com/1471-2296/15/54/prepub
